# Japan's Academic Barriers to Gender Equality as Seen in a Comparison of Public and Private Medical Schools: A Cross-Sectional Study

**DOI:** 10.1089/whr.2021.0095

**Published:** 2022-01-31

**Authors:** Natsuko Nagano, Takashi Watari, Yukihisa Tamaki, Kazumichi Onigata

**Affiliations:** ^1^ Department of Radiation Therapy, Shimane University Hospital, Izumo shi, Shimane, Japan.; ^2^ General Medicine Center, Shimane University Hospital, Izumo shi, Shimane, Japan.; ^3^ Postgraduate Clinical Training Center, Shimane University Hospital, Izumo shi, Shimane, Japan.

**Keywords:** academic medicine, gender bias, Japan, private medical school, public medical school

## Abstract

***Background:*** Gender inequalities persist in Japanese academic medicine. Some public medical schools have introduced various types of career support for women physicians, whereas few private schools have. Few studies describe the representation of women at different academic ranks and adequacy of career support in public and private medical schools in Japan.

***Study Design:*** Cross-sectional descriptive study.

***Methods:*** We used publicly available data from the 2018 National Survey on Career Support for Japanese Women Physicians published by the Association of Japanese Medical Colleges in March 2019, which was answered by departments regarding supporting women physicians. Participants represented 51 public and 29 private medical schools in Japan. The proportion of women at academic ranks and career support availability in private and public medical schools were determined using chi-squared test or Fisher's exact test.

***Results:*** The proportion of women in senior ranks was significantly higher in private (28.2%) than in public medical schools (25.4%) (*p* < 0.001). Excluding associate professors, the proportion of professors, lecturers, and assistant professors was significantly higher in private medical schools (3.8% vs. 5.8%, *p* = 0.002; 12.2% vs. 16.0%, *p* < 0.001; 20.5% vs. 29.9%, *p* < 0.001). More public medical schools provided position support and support for other job aspects (43.1% vs. 20.7%, *p* = 0.043; 70.6% vs. 20.7%, *p* < 0.001).

***Conclusions:*** Public medical schools have lower proportions of women in the academic hierarchy but provide more career support than do private medical schools. Further study is needed to reveal the possible causes of this pattern.

## Highlights

- The proportion of women declines dramatically as academic rank increases in Japan, with only 4.7% women at professor level.
- Fewer women are in leadership ranks in public medical schools compared with private despite the former providing various career supports for women so far.
- Tangible supports help women avoid obstacles that interrupt career advancement.

## Introduction

The total number of women entering medical schools in Japan has increased every year, and in 2019, women comprised 34.4% of all students.^1^ However, Japan ranked 121 out of 153 countries in the Gender Gap Index, dropping from 80th position in 2006.^2^ Furthermore, gender disparities in academic ranks largely persist in academic medicine. In Japan, the descending hierarchy in most cases is professor, associate professor, lecturer, and assistant professor. The other members of the faculty do not hold ranks and are considered to be in the same position rank-wise. A study from 2000 reported that, in Japan, women accounted for only 1.7% of professors^3^; in the United States, however, women accounted for 11.9% of full professors in academia.^4^

In surgical medicine, the number of women surgeons at the full professor rank has been increasing year by year, accounting for 9.8% in 2015 in the United States.^5^ Nevertheless, gender inequalities are documented in other dimensions of academic medicine around the world. Women surgeons at assistant and full professor ranks at medical schools in the United States had fewer publications than men, and women assistant professors had lower citation rates than men at the same level.^6^ Another study reported that the salaries of women at associate and full professor levels in U.S. public medical schools were significantly lower than those of men in the same positions.^7^ These studies suggest that gender disparities remain despite the increasing participation of women in academic medicine. However, little is known about recent trends in the representation of women in Japanese academia based on nationwide cross-sectional studies.

In medicine, previous studies have identified several factors that may have caused the underrepresentation of women. Lyu et al. reported that women surgeon specialists with children and responsibility for more than five domestic tasks considered changing careers.^8^ A study of women neurosurgeons in Europe revealed that many perceived they were unequally treated when considering job opportunities and leadership positions and lacked same-gender role models and mentors.^9^ Scully et al. reported that among the young women who took maternity leave, 52.9% experienced the following: an annual loss of $10,000, an obligation to make up for the shifts after maternity leave, the need to compensate for the financial loss in the practice, and the loss of a productivity bonus.^10^ These consequences thus had negative impacts on job satisfaction. A previous Japanese study reported that being married and having children make it difficult to advance to senior ranks and obtain opportunities to study abroad.^11^

Medicine in Japan is no exception regarding the inadequacy of career support. Kaneto et al. reported that the workforce participation rates of Japanese women physicians exhibited a marked decline in their late 20s to 30s, whereas that of men physicians did not show such a decrease.^12^ An alumnae survey from two private medical schools reported that only one-third of women who had resigned returned to full-time work because of the difficulties in balancing work, childbirth, and child-rearing.^13^ Thus, many women physicians struggle to reconcile career advancement and life events.

The Japanese government conceived the “Japan Revitalization Strategy” in 2015, in which it advocated promoting women's participation in society. Under this strategy, government-run projects with a large budget have been started and increasing types of career support have been introduced for women physicians.^14^ Although financial support from the government has been equally open to public and private medical schools, previous research indicates that more public than private medical schools have been actively working to establish various career support systems and some public medical schools introduced and implemented various types of career support,^15–18^ whereas we rarely found similar reports from private medical schools.

To the best of our knowledge, no studies have investigated the representation of women at academic ranks and the adequacy of career support in public medical schools in comparison with private medical schools. The purpose of this study is to identify differences in the actual situation of women physicians and the characteristics of the support provided to them between public and private medical schools in Japan.

### Public and private medical schools in Japan

To briefly outline the major differences between public and private medical schools, the most significant difference is the admission fees and tuition costs. The average admission fee for private medical schools is triple to quadruple the fee for public medical schools and the first-year tuition fee for private medical schools is approximately seven times more than public medical schools. These fees vary among private medical schools, whereas the two types of public medical schools charge the same amount.^19^

Another difference between the two medical schools is the average number of medical students in the class of 2019. Private medical schools have an average of 117.7 students per class year, whereas public medical schools have a lower average of 105.0 students.^20^ Furthermore, another difference exists in the research grants awarded to both types of schools. Although not specific to medical schools, the applicants from public schools for Japan's Grants-in-Aid for Scientific Research in 2019 were five times as many as those from private schools. Recent trends show that the ratio of grant winners from private schools has increased, whereas that of public schools has decreased for the past 10 years; the gap between the two has decreased.^21^ Nevertheless, public schools have been the major grant recipients for a long time. Thus, the two types of medical schools in Japan exhibit the characteristic differences.

## Methods

### Study design and data source

This was a cross-sectional descriptive study using publicly available data from the 2018 Survey on Career Support for Japanese Women Physicians, published by the Association of Japanese Medical Colleges (AJMC) in March 2019 (https://ajmc.jp/wp/wp-content/themes/ajmc/documents/pdf/activitiss/gender-committee/jyoseiishi_20190909_00.pdf). Survey administration was entrusted and subsidized by the Ministry of Health, Labour and Welfare as part of a career support project for women physicians in 2019. AJMC is a representative body of the public and private medical schools in Japan, and its members comprise medical school deans and teaching hospital directors.

It established the committee for gender equality promotion based on demand to promote women's participation in medicine as women increasingly enter the medical field, but face difficulties in balancing work and life. The committee has focused on investigating the working situations of women physicians in Japan and actively sharing gender issues that emerge in medical institutions and their possible solutions based on its investigations. This committee was responsible for planning and conducting the 2018 National Survey on Career Support for Japanese Women Physicians and reported the results both online and in a book. According to the committee, the survey objective was to investigate the types of career support currently implemented by medical institutions in Japan to promote career advancement or job retention for women physicians, and to introduce these effective types of support nationwide.

The questionnaire was sent to the deans or hospital directors of 125 medical school hospitals (main and affiliate) and 55 municipal hospitals. Among the total of 180 hospitals, we targeted the 80 main medical schools that also own an associated teaching hospital. For faculty members in Japan, the medical school and hospital are the same. They practice medicine, educate medical students, and engage in medical research. The remaining 100 hospitals that were structurally less academic and educational were excluded from this study to improve generalization of the findings.

### Data collection and processing

As we were not permitted to access the raw questionnaire responses collected from individual medical schools, we used the aggregated data, in which each value was summarized at the level of three types of institutions: public, private, and municipal hospitals. We extracted the public and private medical school summary data. The proportion of women, among all physicians, professors, associate professors, lecturers, assistant professors, and other regular positions consisting of full- and part-time faculty members such as fellows and residents who do not carry the top four titles, was calculated.

We then compared the proportion of women in these positions in public medical schools with that of private medical schools. As the survey included various types of support for women offered by several medical schools, we classified them into the following six categories: (1) career advancement and research support, (2) working environment, (3) childcare, (4) mentorship and peer support, (5) financial aid, and (6) back-to-work support after maternity leave. A comparison analysis was performed to investigate whether public or private medical schools were more likely to provide each type of support.

### Statistical analyses

Chi-squared test or Fisher's exact test was used to compare nominal variables. All analyses were performed using Stata statistical software, version 14.0 (Stata Corp. 2015, Stata 14 Base Reference Manual, College Station, TX). All tests were two-sided with *p* < 0.05 considered statistically significant.

### Statement of ethics approval

The survey data we used in our study were publicly disclosed by AJMC and are downloadable on the AJMC website with unrestricted access. To use the data, authors must receive permission from the Ministry of Health, Labour and Welfare through AJMC, as we did. The analysis of the data we extracted from the survey does not render the data individually identifiable. Therefore, our study did not require Institutional Review Board approval.

## Results

### Participant characteristics

**FIG. 1.**
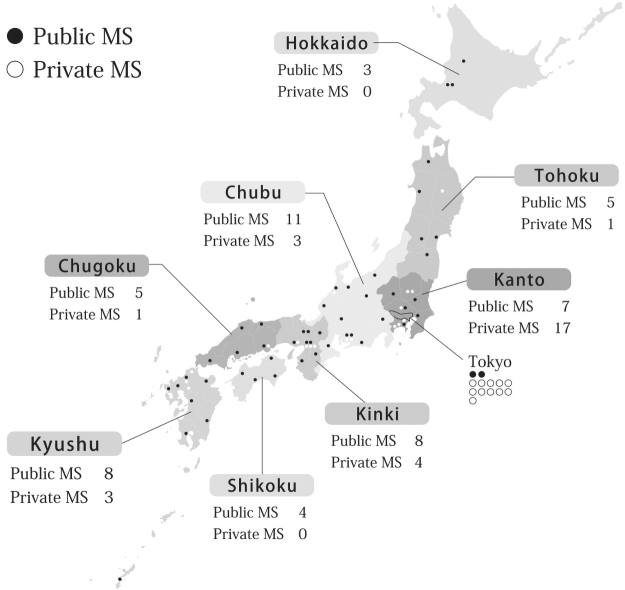
Mapping of 51 public and 29 private medical schools in Japan, 2019. MS, medical schools.

The questionnaire was collected from 51 public medical schools and 29 private medical schools in Japan. Responses were obtained from all schools for a response rate of 100% (Table 1). All existing medical schools were covered except two recently established private medical schools that were not included because they were not members of AJMC. The percentage of women in public and private medical schools was 25.4% and 28.2%, respectively, representing a significant difference (*p* < 0.001). The survey defined prefectures that contained cities with >500,000 inhabitants as medium to large, and those with <500,000 inhabitants as small. Table 1 demonstrates the tendency of a high number of private medical schools to be located in medium to large cities and more public medical schools in small cities. By region, private medical schools were concentrated in the Kanto region, including the Tokyo metropolitan area, whereas public medical schools were evenly spread throughout other local regions (Fig. 1).

**Table 1.** Characteristics of Participants from 80 Medical Schools in Japan, 2019

Characteristics	Public	Private
Number of medical schools	51	29
Number of physicians	33,009	26,142
Men (%)	24,640 (74.6)	18,768 (71.8)
Women (%)	8,369 (25.4)	7,374 (28.2)
Number of medical schools located in large to medium cities	22	25
Number of medical schools located in small cities	29	4
Number of medical schools located in each region		
Hokkaido	3	0
Tohoku	5	1
Kanto	7	17
Chubu	11	3
Kinki	8	4
Chugoku	5	1
Shikoku	4	0
Kyushu	8	3

**FIG. 2.**
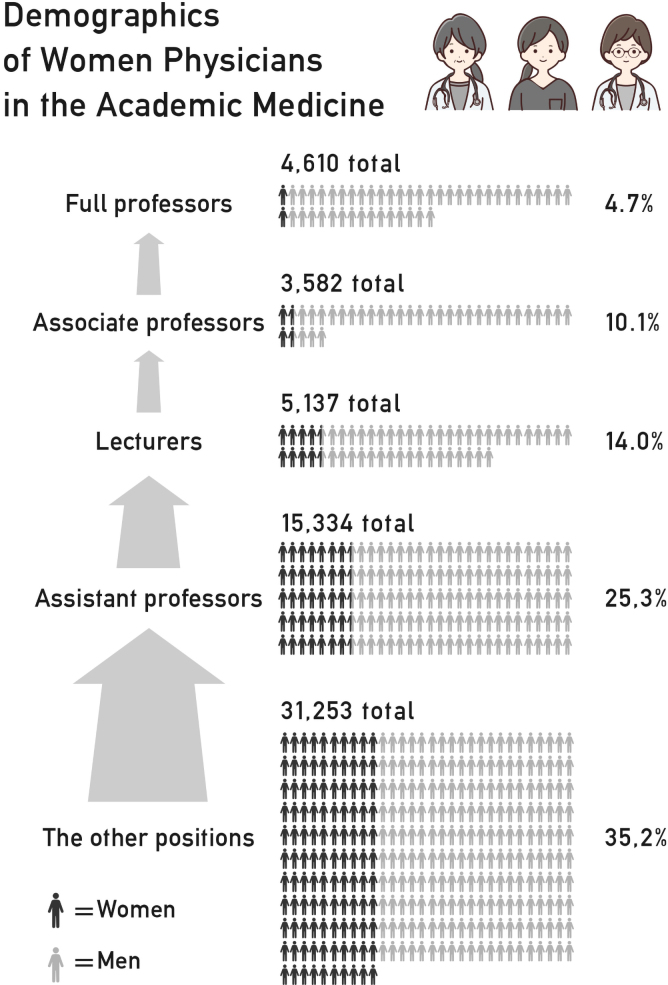
Demographics of women physicians in academic medicine in Japan, 2019. The other positions included full- and part-time women physicians, fellows, and residents not holding the academic ranks.

The proportion of women declined dramatically as academic rank increased (Fig. 2). The proportion of other regular positions including full- and part-time physicians who did not hold any of the four most senior titles accounted for 35.2% (*n* = 10,999) of the sample. However, as the rank increased, the proportion of women decreased and ultimately accounted for only 4.7% at the full professor rank.

### Comparison of academic hierarchy in public and private medical schools

**FIG. 3.**
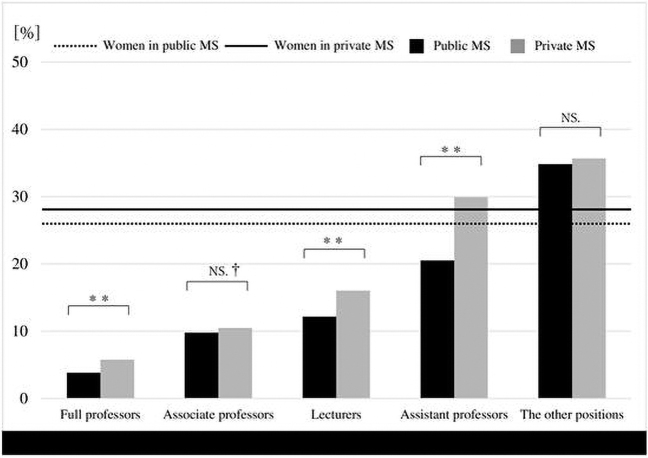
Comparison of public and private medical schools in terms of leadership positions in Japan, 2019. ^†^Not significant; ^**^Significant. The other positions included full- and part-time women physicians, fellows, and residents not holding the academic ranks. MS, medical schools.

Among all of the academic ranks in medical schools, the proportion of women professors, lecturers, and assistant professors was significantly lower in public medical schools than in private medical schools (3.8% vs. 5.8%, *p* = 0.002; 12.2% vs. 16.0%, *p* < 0.001; 20.5% vs. 29.9%, *p* < 0.001, respectively) (Fig. 3). The proportion of women associate professors did not significantly differ between the two medical school groups, although private medical schools had a higher proportion (9.8% vs. 10.5%, *p* = 0.491). Although it is not shown in Figure 3, the overall proportion of professors, associate professors, lecturers, and assistant professors who were women was significantly higher in private medical schools (14.7% vs. 21.6%, *p* < 0.001), whereas the proportion of women in other regular positions was not significantly different between the two groups (34.8% vs. 35.7%, *p* = 0.123).

### Comparison of career support in public and private medical schools

Significantly more public medical schools than private medical schools had more than one department dedicated to offering career support, especially for women (96.1% vs. 79.3%, *p* = 0.016) (Table 2). Both preparing positions for women and other types of support and not offering positions that promote career advancement and research support were significantly higher in public medical schools than in private medical schools (43.1% vs. 20.7%, *p* = 0.043; 70.6% vs. 20.7%, *p* < 0.001). In the category of working environment support, there were significant differences in the availability of working part-time and having no night shift duties between public medical schools and private medical schools (84.3% vs. 17.9%, *p* < 0.001).

**Table 2.** Comparison of Types of Career Support in Public and Private Medical Schools in Japan, 2019

	Public (***n*** = 51)		Private (***n*** = 29)		
Variables	%	***n***	%	***n***	***p***
Designated departments for women's career support	96.1	49/51	79.3	23/29	0.016
(1) Career advancement and research support					
Exclusive positions for women	43.1	22/51	20.7	6/29	0.043
Other advantages (except offering positions)	70.6	36/51	20.7	6/29	<0.001
(2) Working environment					
Regular employment available for shorter working time than full-time	70.6	36/51	78.6	22/28^a^	0.442
Part-time	84.3	43/51	17.9	5/28^a^	<0.001
Job sharing	7.8	4/51	0	0/28^a^	0.333
No night shift duty	86	37/43^a^	60.7	17/28^a^	0.015
No weekend shifts	54.9	28/51	50.0	14/28^a^	0.676
No overtime work	82.4	42/51	64.3	18/28^a^	0.072
Shift-working system	17.6	9/51	14.3	4/28^a^	0.963
Multiple primary physicians	29.4	15/51	21.4	6/28^a^	0.442
(3) Childcare					
Locum physicians during maternity leave	70.6	36/51	34.5	10/29	0.002
Childcare facilities	100	51/51	82.8	24/29	0.010
24-hour childcare	60.8	31/51	37.5	9/24^a^	0.059
Care available on Saturdays	86.3	44/51	91.7	22/24^a^	0.800
Care available on Sundays	33.3	17/51	8.3	2/24^a^	0.033
Care available on holidays	39.2	20/51	4.2	1/24^a^	0.002
Care of sick children	72.5	37/51	66.7	16/24^a^	0.602
Care of post-sick children	84.3	43/51	45.8	11/24^a^	0.113
(4) Mentorship and peer support					
Matching a mentor	31.4	16/51	10.3	3/29	0.057
Peer support	5.9	3/51	3.4	1/29	>0.999
Assistance service	47.1	24/51	37.9	11/29	0.429
(5) Financial aid					
Research	33.3	17/51	6.9	2/29	0.012
Training	9.8	5/51	0	0/29	0.195
Child-rearing	9.8	5/51	0	0/29	0.195
(6) Back-to-work support after maternity leave					
Educational support	25.5	13/51	13.8	4/29	0.346
Recruiting support	7.8	4/51	0	0/29	0.316

^a^ Missing data: “No night shift duty” was missing eight responses for public medical schools. All variables in “(2) Working environment” were missing one response for private medical schools. All variables in “(3) Childcare,” except “locum physicians during maternity leave” and “Childcare facilities” were missing five responses for private medical schools.

Other comparisons revealed no significant differences; however, overall, public medical schools were more likely to provide career support to women than were private medical schools. Regarding the support for childbirth and child-rearing, locum physicians for maternity leave were more likely to be provided by public medical schools than private medical schools (70.6% vs. 34.5%, *p* = 0.002). Childcare facilities for faculty members' children were available at all public medical schools, whereas only 82.8% of private medical schools provided the same service (*p* = 0.010). Significantly more public medical schools extended their childcare services on Sundays and other holidays compared with private medical schools (33.3% vs. 8.3%, *p* = 0.033; 39.2% vs. 4.2%, *p* = 0.002, respectively).

Most other childcare services, such as 24-hour childcare and care of sick and post-sick children, were more accessible at public medical schools. The availability of financial aid for women researchers was higher in public medical schools than private medical schools (33.3% vs. 4.2%, *p* = 0.002), as only a few of the public medical schools offered financial aid for training and child-rearing. Back-to-work support after maternity leave consisted of educational and recruiting support, both of which were more likely to be offered by a public medical school than a private medical school. However, there were missing data in the comparison analysis in Table 2; the survey report did not mention why most missing data were from private medical schools. “No duty of night shifts” was missing eight responses for public medical schools.

All variables in “(2) Working environment” were missing one response for private medical schools. All variables in “(3) Childcare,” except “locum physicians during maternity leave” and “childcare facilities,” were missing responses for five private medical schools. Overall, Table 2 demonstrates that public medical schools were more likely than private medical schools to offer various types of support for women.

## Discussion

Our results demonstrated that there were fewer and fewer women representing academic ranks as they moved up the career ladder. This trend supports a previous cross-sectional study conducted at a single public medical school in Japan, which reported fewer women surgeons in senior positions than their male counterparts as rank increased.^22^ Considering that our study targeted nearly the total population of interest, our results strongly suggest the gender differences persisted in academic medicine.

However, our results shown in Figure 2 might be biased by the decreasing proportion of women physicians of advanced age. In fact, it was reported in 2018 that women physicians <29 years old, in their 30s, 40s, 50s, 60s, and >70 years old accounted for 35.9%, 31.2%, 26.3%, 16.6%, 10.9%, and 9.6% of physicians, respectively.^23^ Thus, it is difficult to determine from the cross-sectional nature of this study whether female leaders have been increasing in number at the same rate as male leaders. To examine this issue, a longitudinal study would be needed.

We also found fewer women in the top four ranks in public medical schools than private medical schools. As no previous study has compared public and private medical schools, and few statistics are available that separately evaluate the two types of medical schools based on gender issues, it is difficult to further discuss the reason for the results in Figure 3. Nevertheless, we explore several possible explanations for our findings, as follows. First, the process, criteria, or requirements for promotion to higher positions could be different between groups. Second, the prepared number of positions for the various academic ranks could be higher in private medical schools. Further studies need to take these possible confounds into account.

Our findings also indicated that private medical schools provided less career support of nearly all types, with some types showing a significant difference compared with public medical schools. Again, the lack of previous studies makes further discussion difficult, although we suggest some potential explanations, as follows. Given that the tuition costs of private medical schools are much higher than those of public medical schools, it is possible that the personal or family wealth of physicians could influence the career support comparison. In addition, nationwide surveys conducted by two ministries of government in Japan showed that extended families tend to live in rural regions, whereas nuclear families and more dual-income households tend to live in urban regions^24,25^

Considering these demographic tendencies, women physicians working in rural regions where public medical schools are more predominant than private medical schools may receive greater support from their own family or neighboring communities and, therefore, need less support from their workplace. Moreover, the percentage of women who actually used each type of career support was uncertain because the survey queried only the availability of types of career support in each medical school. Fujimaki et al. reported that women neurosurgeons showed less recognition of career support systems than did chiefs of neurosurgical departments^26–28^; hence, there is the possibility that few women use the career support or take advantage of them. These factors at the individual level might ultimately have influenced the difference between the two types of institutions.

There was a contradictory finding that private medical schools provided fewer types of career support, but more women were present within the academic ranks. However, given the descriptive nature of the study, we are unable to imply any causal relationship between the findings. Furthermore, it might be premature to conclude that these differences are fixed because it is still unclear whether the various types of career support listed in Table 2 currently satisfy the needs of women physicians. The career support system was initiated in 2015 under a government-led strategy and has been rapidly accommodated by many medical institutions to date. Women physicians currently in their 30s to 40s are expected to benefit most from such support, although physicians already with senior ranks, especially the professor level, have not benefitted. Thus, we need to reassess the situation after a longer period of time has elapsed and conduct observational studies to detect any possible links between the two findings.

### Limitation

Despite its strengths, this study has some limitations. First, although it was a nationwide study in Japan, it cannot be generalized to other countries. In addition, the issue of gender bias is always closely linked to religion, cultural and social background, or government policy, which makes it difficult to conduct quantitative research. Second, we could not include the crucial raw data for multiple regression analysis to elucidate the potential reasons for these complicated problems. As a next step, further quantitative research is necessary to explore the fundamental causes for these problems. However, to the best of our knowledge, this is the first study to described in detail and compare career support barriers among Japanese woman physician between public and private medical schools using the national survey on Career Support for Japanese Women Physicians, and we believe this study will be fundamental as a pilot study in Japan.

## Conclusion

This nationwide cross-sectional descriptive study demonstrated that (1) the proportion of women physicians decreases with increasing academic rank, (2) more women working at private medical schools are present within the academic ranks than at public medical schools, and (3) public medical schools provide more types of career support than do private medical schools. Our data covering most medical schools in Japan showed that gender gaps are still largely present in academia. This was especially remarkable at the professor level. In the comparison of public and private medical schools, public medical schools provided more career support for women. In contrast, fewer women were present at higher positions of all levels. It is yet unclear whether sufficient career support would help women physicians in their career advancement in academic medicine. These results may foster further explorations to evaluate the effect of career support on the representation of women in academic medicine.

## Data Sharing

Data that support the findings of this study are available in the 2018 National Survey on Career Support for Japanese Women Physicians on the homepage of the Association of Japanese Medical Colleges at https://ajmc.jp/wp/wp-content/themes/ajmc/documents/pdf/activities/gender-committee/jyoseiishi_20190909_00.pdf [accessed December 26, 2021, 1-3-11 Yushima Bunkyo-ku, Tokyo 113-0033, TEL: 03-3813-4610].

## Abbreviations Used

AJMC: Association of Japanese Medical Colleges
MS: medical schools
